# Telomere Length Differently Associated to Obesity and Hyperandrogenism in Women With Polycystic Ovary Syndrome

**DOI:** 10.3389/fendo.2021.604215

**Published:** 2021-05-14

**Authors:** Mariela Edith Velazquez, Andrea L. Millan, Mailén Rojo, Giselle Adriana Abruzzese, Silvina Ema Cocucci, Andrea Elena Iglesias Molli, Gustavo Daniel Frechtel, Alicia Beatriz Motta, Gloria Edith Cerrone

**Affiliations:** ^1^ Laboratorio de Fisio-Patología Ovárica, Centro de Estudios Farmacológicos y Botánicos, Consejo Nacional de Investigaciones Científicas y Técnicas, Facultad de Medicina, Universidad de Buenos Aires, Buenos Aires, Argentina; ^2^ Universidad de Buenos Aires, Departamento de Microbiología, Inmunología, Biotecnología y Genética, Facultad de Farmaciay Bioquímica, Cátedra de Genética, Buenos Aires, Argentina; ^3^ CONICET-Universidad de Buenos Aires, Instituto de Inmunología, Genética y Metabolismo (INIGEM), Laboratorio de Diabetes y Metabolismo, Buenos Aires, Argentina

**Keywords:** telomere length, obesity, metabolic and endocrine disorders, hyperandrogenism, polycystic ovary syndrome

## Abstract

**Background:**

Polycystic Ovary Syndrome (PCOS) often present metabolic disorders and hyperandrogenism (HA), facts that may influence the telomere length (TL).

**Aims:**

To compare the absolute TL (aTL) between women with PCOS and control women, and their association with the presence of obesity and HA parameters.

**Materials and methods:**

The PCOS group included 170 unrelated women outpatients and the control group, 64 unrelated donor women. Anthropometric, biochemical-clinical parameters and androgen profile were determined. The PCOS patients were divided accordingly to the presence of obesity and androgenic condition. The aTL was determined from peripheral blood leukocytes by Real Time quantitative PCR.

**Results:**

Women with PCOS exhibited a significantly longer aTL than controls after age adjustment (p=0.001). A stepwise multivariate linear regression in PCOS women, showed that WC (waist circumference) contributed negatively (b=-0.17) while testosterone levels contributed positively (b=7.24) to aTL. The non-Obese PCOS (noOB-PCOS) presented the longest aTL when compared to controls (p=0.001). Meanwhile, the aTL was significantly higher in the hyperandrogenic PCOS phenotype (HA-PCOS) than in the controls (p=0.001) and non hyperandrogenic PCOS phenotype (NHA-PCOS) (p=0.04). Interestingly, when considering obesity and HA parameters in PCOS, HA exerts the major effect over the aTL as non-obese HA exhibited the lengthiest aTL (23.9 ± 13.13 Kbp). Conversely, the obese NHA patients showed the shortest aTL (16.5 ± 10.59 Kbp).

**Conclusions:**

Whilst a shorter aTL could be related to the presence of obesity, a longer aTL would be associated with HA phenotype. These findings suggest a balance between the effect produced by the different metabolic and hormonal components, in PCOS women.

## Introduction

Polycystic ovary syndrome (PCOS), one of the most common gynecological endocrine diseases, affects 6%–20% of women in their reproductive age (1). Its clinical presentation is heterogeneous, and, according to the Rotterdam criteria, clinically defined by the presence of two or more of the following features: oligo- or anovulation, clinical and/or biochemical hyperandrogenism (HA), and polycystic ovarian morphology (2). In this way, the Rotterdam criteria defines four phenotypes, three of which present HA (HA-PCOS phenotypes) and one normo androgenic phenotype (NHA-PCOS phenotype) (2, 3). PCOS is also closely related to metabolic disorders such as insulin resistance, dyslipidemia and obesity (4). In particular, obesity worsens the reproductive and metabolic features in PCOS women through increased insulin resistance and inflammation (5). In this way, obesity promotes dyslipidemia and anovulation associated with PCOS (6). Consequently, the PCOS women with obesity have a more severe phenotype compared to non-obese PCOS (7).

Telomeres are nucleic-protein complexes at the ends of eukaryotic chromosomes, composed of repeats in a hexanucleotide of non-coding DNA (TTAGGG in mammals) and proteins, which play an essential role in the integrity of the chromosome, constituting a critical factor for cell survival (8). In the process of cell division, DNA replicates and TL may have a reduction of about 20 to 40 kilo base pairs (Kbp) per year, in peripheral blood leukocytes. So, TL and age are negatively correlated, with TL decreasing as the age of an individual increases (9–11). Telomeric shortening constitutes an important marker of the replicative capacity of a cell and thus a marker of cellular aging (12). To maintain telomeres, some cells express telomerase, a specialized reverse transcriptase, capable of adding DNA repeats to the 3’ end of the telomeric leader chain by using an RNA molecule as a template (13). Importantly in turn, elevated levels of androgens are related to increased telomerase activity, so HA can positively affect TL (14–16). Additionally, there is an association between obesity and telomere length (TL) since the state of chronic systemic inflammation promoted by obesity induces shortening of the telomere (9). Women with PCOS often presented both metabolic and hormonal abnormalities that may influence the TL (14, 17–20). In that context, although the effect of obesity and HA on TL have been demonstrated separately, the joint effect of HA and obesity has not yet been investigated. Therefore, in the present study we hypothesized that in PCOS patients, the HA can compensate for the negative effect of metabolic abnormalities on absolute telomere length (aTL). Thus, we analyzed the effect of HA and obesity on TL in PCOS women.

## Materials and Methods

### PCOS Patients and Control Women

We estimate the sample size according to a previous report which informed a relative telomere length (ln T/S ratio) was higher in PCOS patients than in the control participants, 2.74 ± 0.19 and 2.54 ± 0.13 respectively (21). These main outcomes were considered in the determination of the effect size of 1.21 (delta). Using Gpower v3.1.9.7, under allocation ratio of 3:1 case-control, a total sample size of 44 individuals was considered sufficient, assuming an alpha level of p = 0.05 and a power of 90%.

We carried out a retrospective study with 170 PCOS patients, unrelated women recruited from attending the Endocrine Division of the Hospital Durand, Buenos Aires, Argentina, from 2006 to 2016. The samples were conserved in adequate condition of temperature minimizing the times stock DNA was freeze-thawed. Diagnosis of PCOS was based on the revised 2003 Rotterdam criteria (2). Patients were excluded if any of the following diseases or conditions were present: history of gestational diabetes, hyperprolactinemia, hepatic or hematological disease, Cushing’s syndrome, 21-hydroxylase deficiency, thyroid dysfunction, or diabetes.

The control women included 64 unrelated blood donors’ women recruited from the Department of Hemotherapy, from 2006 to 2016. All volunteers self-reported no clinical components of PCOS or familiar history of PCOS, normal menstrual cycles, and had normal findings in medical examination and blood counts.

Control women showed none of the exclusion criteria and no other associated pathologies. None of the participants had received any hormonal or insulin-modifying therapy for at least 2 months before the study or any other therapies that could affect the metabolism or reproductive system. Exclusion criteria included other pituitary, adrenal or ovarian diseases.

All the patients and the control group were Argentinean, and all filled out an informed written consent. The study was conducted in accordance with the 1964 Helsinki Declaration and its later amendments or comparable ethics standards and approved by the local research Institutional committees of the School of Medicine and School of Biochemistry, University of Buenos Aires; and Hospital Carlos G. Durán, Buenos Aires, Argentina.

### Clinical Measurements

Participants provided their age. Anthropometric measurements, including height, weight and waist circumference (WC) and body mass index (BMI) score, were determined by standardized protocol in every subject. WC was measured at the end of a normal expiration, as the narrowest circumference of the trunk with an inelastic fiberglass standard tape over the unclothed abdomen, at the narrowest point between the costal margin and iliac crest. Systolic and diastolic blood pressure (SBP and DBP respectively) were recorded using a standard mercury sphygmomanometer after at least 10 min of rest. After a 12-h overnight fast, fasting blood samples were drawn from every individual at 8 a.m., during early follicular phase (days 1-5 of the menstrual cycle for eumenorrheic or oligomenorrheic patients, or at any moment for amenorrheic patients). Total cholesterol (TC), triglycerides (TG), low-density cholesterol (LDL-C), high-density lipoprotein cholesterol (HDL-C), glucose, and insulin were measured in serum on a Cobas 8000 autoanalyzer (Roche). Insulin was determined by chemiluminescence on a Liaison instrument (Diasorin) with intra- and inter-assay coefficients of 4.3% and 11.6%, respectively. Homeostasis Model Assessment (HOMA) was calculated with the following formula: (basal glucose mg/dL x basal insulin µUI/mL)/405. The Quantitative Insulin Sensitivity Check index (QUICKI) was calculated as follows: 1/(log basal insulin μU/mL] + log basal glucose mg/dL).

The androgen profile, including total testosterone, androstenedione, dehydroepiandrosterone-sulfate (DHEA-S) and 17α-hydroxyprogesterone (17OHP), SHBG and the LH/FSH ratio was evaluated in serum to identify PCOS patients. Total testosterone was measured by a chemiluminescence assay on a Beckman-Coulter Access 2 analyzer, whereas 17OHP and androstenedione were measured with a commercial radioimmunoassay (RIA-CT, DIASource) and DHEA-S by an Immulite 1000 (DPC) kit. LH and FSH were measured by chemiluminescence on Access analyzer and SHBG by chemiluminescence Immulite (22). The coefficients of variation for FSH, LH and SHBG were 7.72%, 6.54% and 9.93% respectively.

### PCOS Phenotype Assessment

The PCOS patients were divided into two phenotypes: HA-PCOS and NHA-PCOS (when HA data was available). The patients were considered HA-PCOS by the presence of clinical and/or biochemical signs of androgen excess as previously reported (22). The patients were considered NHA-PCOS when no clinical or biochemical HA was present. Additionally, two subgroups were identified in PCOS women according to the presence of obesity (BMI ≥ 30.0 kg/m2) (when BMI data was available): obese PCOS women (OB-PCOS) and non-obese PCOS (noOB-PCOS) (23).

Also, the PCOS patients were divided considering the presence or absence of obesity and androgenic condition, therefore four subgroups were determined: non-obese non hyperandrogenic PCOS (noOB-NHA), non-obese hyperandrogenic PCOS (noOB-HA), obese non hyperandrogenic PCOS (OB-NHA) and obese hyperandrogenic PCOS (OB-HA).

### Measurement of Absolute Telomere Length

Venous blood samples from each participant were collected between 7 AM and 9 AM, whole blood (anticoagulated with EDTA) was stored at −20°C for later assessment. Genomic DNA samples were isolated from peripheral blood leukocytes by the CTAB technique, and aTL was determined based on a Real-Time PCR protocol previously described (24, 25). Briefly, the length of the repetitive sequences of telomeres (Kbp of telomeric sequence/reaction, T) compared with a single copy gene (large ribosomal protein P0 subunit, RPLP0 gene), which represented the genome copies/reaction (S), were determined by standard curves. The calculation of the T/S ratio allowed the determination of the Kbp of telomeric sequence per cell for each individual. The PCR reactions were performed in a StepOne ™ Real-Time PCR System (Applied Biosystems, California, USA) in duplicate for all the samples studied. DNA samples were then diluted with sterile nuclease-free water (Invitrogen, Waltham, MA, USA) to a concentration of 10 ng/μL and stored at -20°C for up to two weeks. The DNA (20 ng) was amplified in a 20 μL reaction volume containing 10 μL of SYBR Select Master Mix, and 250 nM of primers were added for the RPLP0 gene or 100 nM of primers for the telomeric sequence. RPLP0 gene was amplified using the following primers: forward, 5’- CAGCAAGTGGGAAGGTGTAATCC-3’ and reverse, 5’- CCCATTCTACATCAACGGG TACAA -3’ while the telomeric sequences was amplified using the following primers: forward, 5’- CGGTTTGTTTGGGTTTGGGTTTGGG TTTGGGTTTGGGTT -3’ and reverse, 5’- GGCTTGCCTTACCCTTACCCTTACCCTTACC CAATCCCT -3’. The PCR conditions consisted of a denaturation of 10 min at 95°C, followed by 40 cycles at 95°C for 15 seconds, 60°C for 1 minute and the Melting curve: 1 cycle of 15 seconds at 95°C, 1 minute at 60°C and 15 seconds at 98°C, with a temperature ramp of 0.3°C/second.

### Statistical Analysis

Descriptive analysis was performed, and quantitative data were analyzed using the Statistical Package for Social Sciences (SPSS version 20). Normal distribution of the data was assessed by the Shapiro-Wilk test. Characteristics of PCOS patients and control groups with a normal distribution were represented as mean ± standard error of mean (SEM) and compared by unpaired two-tailed Student’s test. When the normalization was not possible, the characteristics were expressed as median and interquartile range and the Mann Whitney test for skewed data was used to test differences between groups. Univariate analysis of covariance adjusting for age (ANCOVA), followed by Bonferroni post-hoc test was applied for multiple factors or groups. The association between aTL and quantitative variables was analyzed by partial correlation analysis, adjusted by the effect of age. A stepwise regression analysis was performed to jointly consider the variables that significantly contribute to aTL. p values lower than 0.05 were considered as statistically significant for each test.

## Results

### Phenotype Characterization

The biochemical-clinical characteristics of the PCOS patients and control group are shown in **Table 1**. Compared to controls, PCOS patients had higher BMI, weight, WC, and higher levels of TC, LDL-C, TG, Glucose, Insulin, HOMA-IR and lower levels of QUICKI. These differences remained significant after adjusting for age. There were no differences in age, SBP and DBP.

**Table 1:** Comparison of biochemical and clinical characteristics between control groups and PCOS patients.

Features	control (n=64)	PCOS (n=170)	p control vs. PCOS	p control vs. PCOS*
**Age (years)**	26.80 ± 0.54	26.16 ± 0.36	0.35	
**aTL (Kbp)**	14.54 ± 1.04	18.78 ± 0.94	**0.003**	**0.001**
**BMI (kg/m^2^)**	22.02 ± 0.32	31.31 ± 0.64	**<0.001**	**<0.001**
**Weight (kg)**	60.60 ± 0.80	79.88 ± 1.76	**<0.001**	**<0.001**
**WC (cm)**	74.77 ± 1.04	95.22 ± 1.46	**<0.001**	**<0.001**
**SBP (mmHg)**	110 (110-120)	110 (100-120)	0.08	0.09
**DBP (mmHg)**	70 (70-80)	70 (70-80)	0.21	0.16
**TC (mg/dL)**	159.57 ± 3.04	188.07 ± 3.49	**<0.001**	**<0.001**
**HDL-C(mg/dL)**	52.79 ± 1.67	50.59 ± 1.28	0.27	0.39
**LDL-C (mg/dL)**	88.60 (88.60-105.10)	114.70 (93-140)	**<0.001**	**<0.001**
**TG (mg/dL)**	70.70 ± 3.57	121.71 ± 5.39	**<0.001**	**<0.001**
**Glucose (mg/dL)**	81.69 ± 1.08	89.69 ± 1.01	**<0.001**	**<0.001**
**Insulin (mg/dL)**	7.61(7.61-11.12)	13.70(8.70-21)	**<0.001**	**<0.001**
**HOMA-IR**	1.64 (1.64-2.35)	3.92 (1.84-4.64)	**<0.001**	**<0.001**
**QUICKI**	0.36 ± 0.004	0.33 ± 0.003	**<0.001**	**<0.001**

Values are expressed as mean ± SEM or median and 25-75 interquartile range (Test U-Mann Whitney). p value: unpaired two-tailed Student’s test or Mann-Whitney depending on the equality of the variance or not, respectively. p value* adjusted by age (Multiple linear regression) patients. p<0.05 was considered as significant and highlighted in bold. aTL, absolute telomere length; BMI, body mass index; WC, waist circumference; SBP, systolic blood pressure; DBP, diastolic blood pressure; TC, total cholesterol; HDL-C, high density cholesterol; LDL-C, low density cholesterol; TG, triglycerides; HOMA-IR, homeostasis model assessment of insulin resistance; QUICKY, Quantitative Insulin Sensitivity Check Index.

### aTL Study

**Figure 1:**
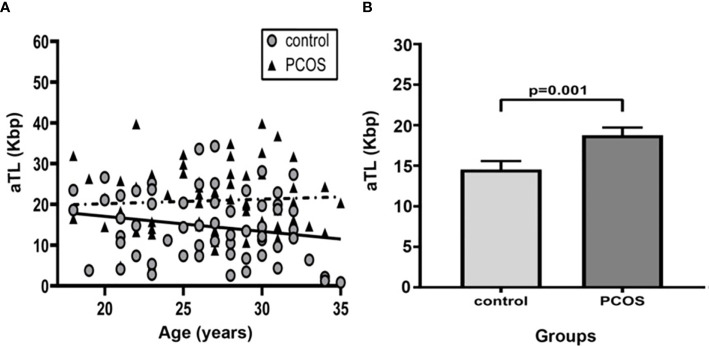
Comparison of Absolute telomere length between control group and PCOS. **(A)** Distribution of absolute telomere length according to age in control and PCOS groups. (—): The linear regression for control group (R^2 =^ 0.15; Regression coefficient r=-0.28, p=0.25) and (- - -): PCOS group (R^2 =^ 0.18; Regression coefficient r=-0.46; p=0.02). **(B)** Bar graphs illustrates mean ± standard error of mean (SEM) of aTL in control and PCOS groups. Univariate analysis adjusted by age (ANCOVA); p<0.05 was considered as significant. aTL, absolute telomere length; Kbp, kilobase pairs; PCOS, polycystic ovary syndrome.

The aTL distribution related to age in control and PCOS groups is shown in **Figure 1A**. There was an expected decline in aTL with increasing age for all studied women (Regression coefficients r=-0.44; R^2 =^ 0.18; p=0.006; regression not shown).

In comparing the mean aTL between groups, individuals with PCOS exhibited a significantly longer aTL than controls after adjusting for age (18.78 ± 12.25 Kbp vs. 14.54 ± 8.29 Kbp, p=0.001) (**Figure 1B** **)**. After adjusting for age and BMI the mean aTL remained significantly different (p=0.003).

We then sought to determine the contribution of clinical and biochemical features to the variability of aTL in PCOS women. A stepwise selection multivariate linear regression showed that WC contributed negatively to aTL (Partial coefficient b=-0.17; p=0.007) while testosterone levels contributed positively (Partial coefficient b=7.24; p=0.04). Together, WC and testosterone significantly accounted for the 9.4% of aTL variability (p=0.002).

**Figure 2:**
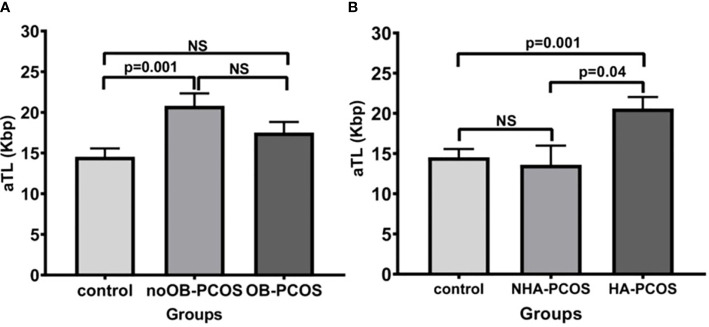
Comparison of absolute telomere length between control group and PCOS according to the presence or absence of obesity **(A)** and hyperandrogenic condition **(B)**. Bar graphs illustrates mean ± standard error of mean (SEM) of absolute telomere length for each group. noOB-PCOS, non-obese PCOS; OB-PCOS, obese PCOS women; NHA-PCOS, non hyperandrogenic PCOS; HA-PCOS, hyperandrogenic PCOS. Univariate analysis adjusted by age (ANCOVA), followed by Bonferroni post-hoc test for multiple factors or groups. NS, non significant difference; p<0.05 was considered as significant; aTL, absolute telomere length; Kbp, kilobase pairs.

The PCOS patients were subdivided into noOB-PCOS (n=72) and OB-PCOS (n=78). A total of 20 PCOS patients could not be classified into any group due to lack of BMI registration and were not included in this section of the study. In the univariate analysis adjusted by age, the PCOS condition and also the obesity contribution to aTL lengthening was observed, as noOB-PCOS presented the longest aTL when compared to healthy controls (20.7 ± 12.9 Kbp vs. 14.5 ± 8.3 Kbp, p=0.001) (**Figure 2A**).

Meanwhile, the PCOS patients were divided into HA-PCOS (n=80) and NHA-PCOS (n=15). A total of 75 PCOS patients were not analyzed because of the lack of hyperandrogenism parameters. The mean aTL was significantly higher in the HA-PCOS phenotype than in the control group (20.6 ± 12.39 Kbp vs. 14.5 ± 8.31 Kbp, p=0.001) and NHA-PCOS phenotype (13.6 ± 9.28 Kbp, p=0.04) (**Figure 2B**).

**Figure 3:**
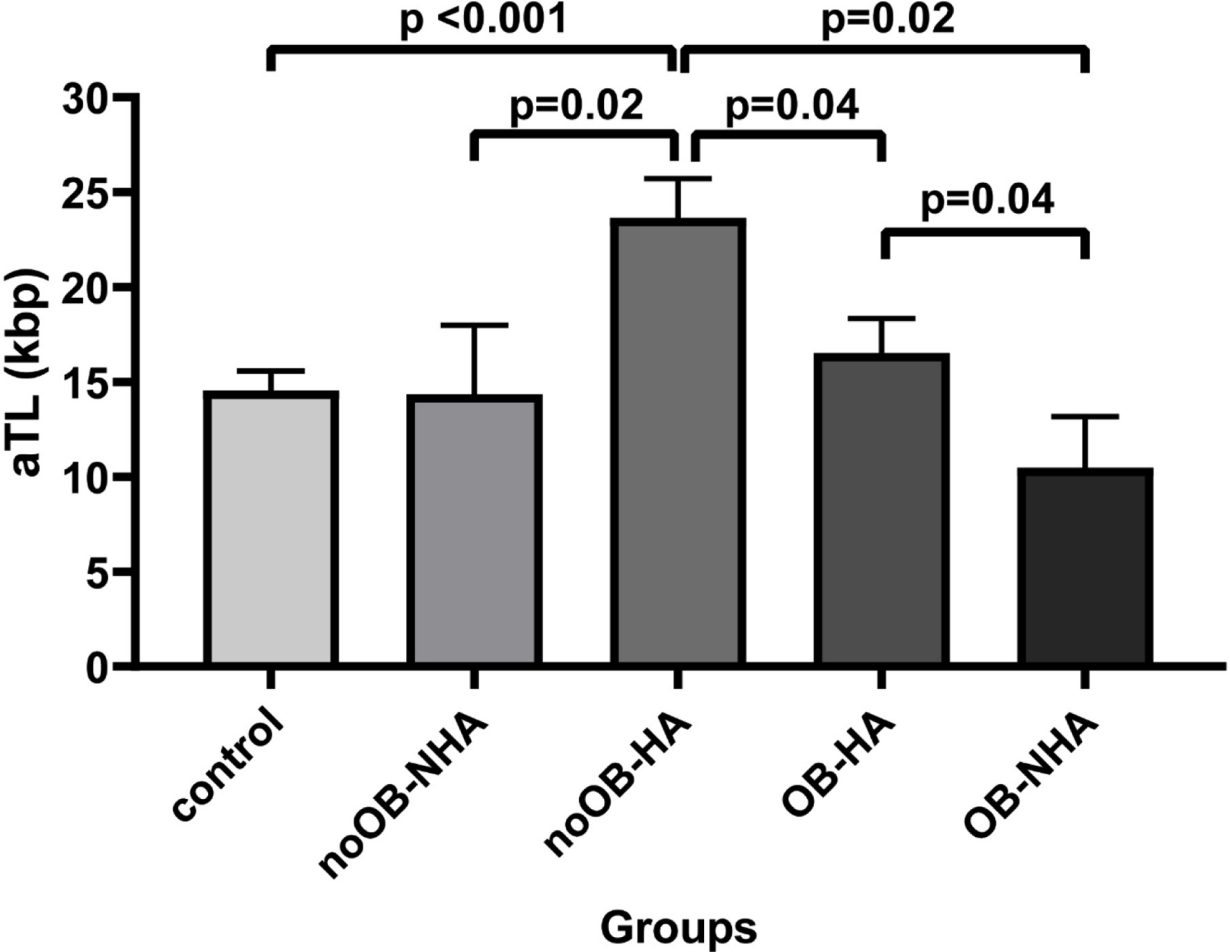
Comparison of absolute telomere length between control and PCOS according to obesity and androgenic condition simultaneously presence. Bar graphs illustrates mean ± standard error of mean (SEM) of absolute telomere length for each group. noOB-NHA, non-obese non hyperandrogenic PCOS; noOB-HA, non-obese hyperandrogenic PCOS; OB-NHA, obese non hyperandrogenic PCOS; OB-HA, obese hyperandrogenic PCOS. Univariate analysis adjusted by age (ANCOVA), followed by Bonferroni post-hoc test applied for multiple factors or groups. aTL, absolute telomere length; p<0.05 was considered as significant; Kbp, kilobase pairs.

Interestingly, **Figure 3** presented the obesity and HA contribution (n=35) to aTL in the PCOS group compared to the control group. HA exerts the major effect over the aTL as noOB-HA (n=7) exhibited the lengthiest aTL (23.9 ± 13.13 Kbp) and differed significantly to all other groups analyzed. Conversely, OB-NHA patients (n=7) showed the shortest aTL (16.5 ± 10.59 Kbp).

## Discussion

PCOS represents one of the most prevalent endocrine pathologies among women of reproductive age, associated with metabolic and hormonal disorders such as obesity, insulin resistance and HA (1, 2). The high prevalence of obesity in women with PCOS exacerbates the hormonal and clinical manifestations, contributing to the development of the disorder (26). Obesity and metabolic syndrome are common metabolic alterations in PCOS patients that may lead to a reduction of the TL through oxidative stress and a chronic inflammation state (9, 27). Furthermore, many studies have focused on the increased expression and activity of telomerase stimulated by androgens, such as testosterone, in ovarian cancer cells and in cultured peripheral blood lymphocytes (14, 15). Also, *in vivo* hormone replacement therapy has been reported to decrease the rate of telomere shortening in postmenopausal women (16). In this sense, the HA condition of women with PCOS would lead to a decreased telomere attrition. Thus, our main objectives were to study the aTL in PCOS patients compared to controls, and to analyze the contribution of obesity and HA.

We observed a negative correlation between aTL and age in the whole population. This result was predicted, considering that age is the main factor involved in the shortening of telomeres and allowed us to validate the method. When we compared the mean aTL between all studied groups, individuals with PCOS exhibited a significantly longer aTL than controls (adjusted for age and BMI), despite the worsened metabolic profile associated with PCOS. We found that in the PCOS group, both WC and testosterone levels contributed to aTL variability. The WC contributed to the shortening of TL; in addition, the strong positive contribution of testosterone levels would result in the increase of aTL observed in PCOS women.

Previous studies have revealed a controversial relationship between TL and PCOS. Some researches have shown no differences in the TL between PCOS patients and controls. While Wei et al. attribute this result to the narrow age cohort design and limited sample size, Pedroso et al. compare women PCOS and controls with significantly different ages (28, 29). Li et al. reported that women with PCOS showed a shorter aTL than healthy women (range: 13-54 years) and; in agreement with our findings, Wang et al. found that PCOS patients had significantly longer telomeres than control subjects (range: 19-30 years), and showed a positive correlation between aTL and testosterone levels (21, 30). However, the authors do not argue about the effect of metabolic abnormalities on TL within their study. These discrepancies may be attributed to differences in the age ranges analyzed, as we studied young populations ranging between 18 and 35 years of age. We found a significant inverse association between age and aTL in the noOB-PCOS associated with HA, particularly within the metabolically healthy PCOS women (without Metabolic syndrome, groups; data not shown). In addition, our results suggested that the HA condition can compensate for the harmful effect of metabolic abnormalities such as obesity on aTL of women with PCOS (see also **Supplementary Figure 1** in **Supplementary Material**).

It must be considered that TL varies according to the cell type, so it could be useful for future works to determine the TL in granulosa cumulus cells (GC). GC plays an important role in folliculogenesis and steroidogenesis, molecular processes normally altered in PCOS women (31, 32). TL in GC would provide more knowledge about the role of telomeric length in PCOS pathophysiology. In this way, Wei et al. observed significantly lengthened telomeres in granulosa cells while Pedroso et al. showed not altered TL in reproductive cells, both studies were performed in women with PCOS who underwent *in vitro* fertilization/intracytoplasmic sperm injection (IVF/ICSI) fertility treatment (28, 29).

Also, it would be relevant to measure the telomerase activity (TA) in the PCOS patients, due to the role of telomerase in follicular development. In this sense, Li et al. compare TA in GC between PCOS and non-PCOS patients. These authors hypothesized that the abnormal follicular development in PCOS women was not associated with TA, but rather with metabolic disorders and hormonal abnormalities (33).

A recent review observed shorter telomeres and diminished telomerase activity in granulosa cells associated with ovarian insufficiency, so more studies are required to confirm the results (34).

One limitation of the present study is based on the number of patients studied that were subdivided into several groups according to the presence of obesity and androgenic condition (noOB-NHA, noOB-HA, OB-NHA, OB-HA). Although significant differences were observed in mean aTL between the different groups, a greater number of PCOS patients would have increased the power of this study. Furthermore, in the control group it was not possible to measure the impact of hormones on TL due to the lack of clinical hormonal data. In addition, future longitudinal study could provide more information about the variation in TL as a function of other variables.

In conclusion, we corroborate that a balance between the effect of different metabolic and hormonal components such as WC and testosterone respectively, determined the lengthened aTL observed in PCOS group compared to controls. While a shorter aTL could be related to the presence of obesity, a longer aTL would be associated with HA. Taken together, our findings contribute to the knowledge of the pathophysiology of PCOS.

## Data Availability Statement

The datasets analyzed for this study can be found in Figshare.com, https://figshare.com/s/072793261938c34ea9ab.

## Ethics Statement

The studies involving human participants were reviewed and approved by local research Institutional committees of the School of Medicine, School of Pharmacy and Biochemistry of the University of Buenos Aires, and Hospital Carlos G. Durán, Buenos Aires, Argentina. The patients/participants provided their written informed consent to participate in this study.

## Author Contributions

MEV and ALM contributed equally to the manuscript and performed the laboratory work, the data analysis, and the writing. AEIM, SEC, and MR contributed to the telomere determinations. GDF assisted in the design of the research study. GAA, ABM, and GEC contributed to the study design, paper writing, and the final version of the manuscript. All authors contributed to the article and approved the submitted version.

## Funding

This work was supported by grants from Universidad de Buenos Aires (20720160100004BA/2017) to GEC and by Agencia Nacional de Promocion Científica y Tecnológica (PICT 632/ 2016) to ABM. 

## Conflict of Interest

The authors declare that the research was conducted in the absence of any commercial or financial relationships that could be construed as a potential conflict of interest.

## Abbreviations

PCOS, Polycystic Ovary Syndrome; HA, Hyperandrogenism; HA-PCOS, Hyperandrogenic PCOS; NHA-PCOS, Non hyperandrogenic PCOS; TL, Telomere length; aTL, absolute telomere length; PCR, Polymerase chain reaction; WC, waist circumference; Kbp, kilobase pair; BMI, Body mass index; SBP, Systolic blood pressure; DBP, Diastolic blood pressure; TC, Total cholesterol; LDL-C, Low-density lipoprotein cholesterol; HDL-C, High density lipoprotein cholesterol; HOMA, Homeostasis Model Assessment; QUICKI, Quantitative Insulin Sensitivity Check index; DHEA-S, dehydroepiandrosterone-sulfate; 17 OHP, 17α-hydroxyprogesterone; SHBG, Sex hormone binding globulin; LH, Luteinizing hormone; FSH, Follicle stimulating hormone; OB-PCOS, Obese PCOS; noOB-PCOS, Non obese PCOS; CTAB, Cetyl Trimethyl Ammonium Bromide; SEM, standard error of mean; ANCOVA, Analysis of covariance; noOB-HA, Non obese hyperandrogenic PCOS; OB-NHA, Obese non hyperandrogenic PCOS
