# Supplementary material for: Telomere Length Differently Associated to Obesity and Hyperandrogenism in Women With Polycystic Ovary Syndrome
Source: Front Endocrinol (Lausanne). 2021 May 14;12:604215. doi: 10.3389/fendo.2021.604215 (PMC8162376; doi:10.3389/fendo.2021.604215)
Supplement: Supplementary file 1 [file DataSheet_1.pdf]

Supplementary Table1. Clinical and biochemical parameters of the control and PCOS groups divided by androgenic condition.

Features	Control (n=64)	NHA-PCOS (n=15)	HA- PCOS (n=80)	P	p Control vs. NHA*	p Control vs. HA*	p NHA vs HA*
**Age (years)**	26.97±4.19a	27.47±5.52ab	25.24±4.77	**0.03**			
**aTL (Kbp)**	15.19±8.19	12.91±10.63	20.89±13.03	**<0.01**	0.66	**<0.01**	**0.04**
**BMI (kg/m²)**	22.02±2.48a	32.52±7.31b	30.31±7.73b	**0.001**	**0.001**	**0.001**	0.31
**Weigth (kg)**	60.60±5.49a	77.91±20.31b	78.55±20.61b	**0.001**	**0.001**	**0.001**	0.33
**WC (cm)**	74.77±8.47a	98.10±13.36b	92.50±17.38b	**0.001**	**0.001**	**0.001**	0.19
**SBP (mmHg)**	110 (110-120)	110 (110-130)	110 (110-130)	0.32	0.92	0.09	0.08
**DBP (mmHg)**	70 (70-80)	70 (70-80)	70 (70-80)	0.38	0.25	0.09	0.35
**TC (mg/dL)**	159.57±24.19a	201.50±45.84b	185.85±38.66b	**0.03**	**0.001**	**0.001**	0.44
**HDL-C (mg/dL)**	52.79±12.96	52.45±15.51	51.16±13.57	0.60	0.65	0.54	0.83
**LDL-C (mg/dL)**	88.60 (88.6-105.1)a	135.50 (135.50-166.25)b	105.50 (107-137)b	**0.001**	**0.001**	**0.001**	**0.05**
**TG (mg/dL)**	61±28.33a	123.69±54.07b	107.54± 54.33b	**0.001**	**0.001**	**0.001**	0.11
**Glucose (mg/dL)**	82.14±8.66a	92.92±12.01b	89.85±12.15b	**0.001**	**0.001**	**0.001**	0.12
**Insulin (mg/dL)**	7.61(7.61-11.12)a	13.80 (13.80-15.90)b	12.95 (13-21)b	**0.001**	**0.001**	**0.001**	0.89
**HOMA-IR**	1.64 (1.64-2.35)a	2.95(2.95-4.11)b	2.73(2.75-4.78)b	**0.001**	**0.001**	**0.001**	0.73
**QUICKI**	0.36±0.03a	0.32±0.04b	0.33±0.03b	**0.001**	**0.001**	**0.001**	0.65

Values are expressed as mean ± SD (ANOVA and post-hoc Bonferroni) or Median and 25-75 interquartile range (Kruskall Wallis Test). NHA-PCOS: Non hyperandrogenic PCOS phenotype, HA-PCOS: Hyperandrogenic PCOS phenotype, BMI: Body mass index, WC: waist circumference, SBP: Systolic blood pressure, DBP: Diastolic blood pressure, TC: Total cholesterol, HDL-C: High density cholesterol, LDL-C: Low density cholesterol, TG: Triglycerides, HOMA-IR: Homeostasis model assessment of insulin resistance, QUICKY: Quantitative Insulin Sensitivity Check Index. Different letters a and b express statistical difference between groups in univariate analysis. P<0.05 was considered as significant. p value* adjusted by age (ANCOVA) for control vs. PCOS phenotypes and between PCOS subphenotypes.

Supplementary Table 2. Clinical and biochemical parameters of the control and PCOS groups divided by obesity condition.

Features	Control (n=64)	noOB-PCOS (n=72)	OB-PCOS (n=78)	P	p Control vs noOB*	p Control vs OB *	p noOB vs OB*
**Age (years)**	26.97±4.19	25.00 (22.00-30.00)	25.50 (23.00-30.00)	0.133			
**aTL (Kpb)**	14.5±8.3a	20.7±12.9b	17.4±11.3ab	**0.008**	**0.001**	0.354	0.110
**BMI (kg/m^2)**	22.02±2.48a	24.68±3.20b	37.43±5.51c	**<0.001**	**<0.001**	**<0.001**	**<0.001**
**Weigth (kg)**	60.2±5.49a	62.7±10.27a	92.30 (83.60-103.00)b	**<0.001**	0.624	**<0.001**	**<0.001**
**WC (cm)**	74.77±8.47a	82.1±10.32b	107.18±11.91c	**<0.001**	**<0.001**	**<0.001**	**<0.001**
**SBP (mmHg)**	115 (110-120)a	102 (90-120)b	110 (100-130)a	**0.046**	**0.008**	0.407	**<0.001**
**DBP (mmHg)**	70 (70-80)	70 (60-80)	70 (70-80)	**0.009**	**0.004**	0.676	**0.007**
**TC (mg/dL)**	158.79±23.57a	183.50±39.17b	187 (163.25-219.75)b	**<0.001**	**<0.001**	**<0.001**	0.892
**HDL-C(mg/dL)**	52 (44-57)a	56.15±15.31[a]	44 (38-51)b	**<0.001**	0.478	**0.004**	**<0.001**
**LDL-C**	91.85±21.88a	103.50 (88.25-132.75)b	120.21±30.82c	**<0.001**	**0.003**	**<0.001**	0.152
**TG (mg/dL)**	60 (49-94)a	97.00 (66.75-140.75)b	123 (93-176)c	**<0.001**	**<0.001**	**<0.001**	**0.011**
**Glucose**	81.90±8.41a	86 (81-94)b	91.81±12.48c	**<0.001**	**0.003**	**<0.001**	0.107
**Insulin (mg/dL)**	8.56±4.08a	10.60 (5.55-13.30)b	19.00 (13.85-25.78)c	**<0.001**	0.134	**<0.001**	**<0.001**
**HOMA-IR**	1,75±0.87a	2.20 (1.18-3.02)b	4.34 (3.04-5.75)c	**<0.001**	0.409	**<0.001**	**<0.001**
**QUICKI**	0.36 (0.34-0.38)a	0.34 (0.32-0.37)b	0.31 (0.29-0.32)c	**<0.001**	0.123	**<0.001**	**<0.001**

Values are expressed as mean ± SD (ANOVA and post-hoc Bonferroni). Median and 25-75 interquartile range (Kruskall Wallis Test). P value: univariate analysis. Different letters a, b and c express statistical difference between groups in univariate analysis., noOB-PCOS: non obese PCOS women, OB-PCOS: Obese PCOS, aTL: Absolute telomere length, BMI: Body mass index, WC: waist circumference, SBP: Systolic blood pressure, DBP: Diastolic blood pressure, TC: Total cholesterol, HDL-C: High density cholesterol, LDL-C: Low density cholesterol, TG: Triglycerides, HOMA-IR: Homeostasis model assessment of insulin resistance, QUICKY: Quantitative Insulin Sensitivity Check Index. P<0.05 was considered as significant. p value* adjusted by age (ANCOVA) for control vs. PCOS with and without obesity and intra PCOS.

Supplementary Table 3. Clinical and biochemical parameters of the control and PCOS groups divided by metabolic condition.

Features	Control (n=64)	MH-PCOS (n=64)	MetS-PCOS (n=20)	P	p Control vs. MH*	p Control vs. MetS*	p MH vs MetS*
**Age (years)**	26.77±4.36a	23.00 (21.00-29.75)b	29 (23-31)a	**0.007**			
**aTL (Kpb)**	14.32±8.03a	15.96 (9.68-26.94)b	18.82±10.38ab	0.07	**0.008**	0.151	0.442
**BMI (kg/m²)**	22.11±2.56a	28.20 (22.97-34.71)b	34.20±5.49c	**<0.001**	**<0.001**	**<0.001**	**0.028**
**Weigth (kg)**	60.40±5.76a	71.00 (58.55-88.00)b	90.30±15.67c	**<0.001**	**<0.001**	**<0.001**	**0.005**
**WC (cm)**	73.69±7.52a	90.19±16.79b	104.87±11.29c	**<0.001**	**<0.001**	**<0.001**	**0.003**
**SBP (mmHg)**	115 (110-120)	107.5 (90.0-120.0)	117.35±21.22	0.082	**0.023**	0.329	0.052
**DBP (mmHg)**	70 (70-80)	70 (60-80)	70 (70-80)	0.458	**0.017**	0.847	0.196
**TC (mg/dL)**	159.89±24.52a	183.18±39.20b	204.63±40.92c	**<0.001**	**<0.001**	**<0.001**	**0.012**
**HDL-C(mg/dL)**	52 (44-57)a	53.75±12.67a	43.26±10.52b	**0.002**	0.597	**0.002**	**0.003**
**LDL-C (mg/dL)**	91.91±22.06a	102.0 (89.0-137.5)b	131.30±31.82c	**<0.001**	**0.001**	**<0.001**	**0.016**
**TG (mg/dL)**	60.0 (48.5-84.5)a	87.75±30.29b	173.95±59.24c	**<0.001**	**0.001**	**<0.001**	**<0.001**
**Glucose (mg/dL)**	81.85±8.78a	87.00 (81.22-93.00)b	98.70±11.29c	**<0.001**	**0.002**	**<0.001**	**<0.001**
**Insulin (mg/dL)**	8.92±4.37a	11.35 (7.90-18.84)b	19.05±9.16c	**<0.001**	**<0.001**	**<0.001**	0.076
**HOMA-IR**	1.73 (1.09-2.43)a	2.39 (1.43-4.36)b	4.57±2.30c	**<0.001**	**0.001**	**<0.001**	**0.02**
**QUICKI**	0.35 (0.33-0.38)a	0.34 (0.31-0.36)b	0.30 (0.30-0.32)c	**0.003**	**0.005**	**<0.001**	**0.026**

Values are expressed as mean ± SD (ANOVA and post-hoc Bonferroni). Median and 25-75 interquartile range (Kruskall Wallis Test). P value: univariate analysis. Different letters a, b and c express statistical difference between groups in univariate analysis. MH-PCOS: Metabolically Healthy PCOS, MetS-PCOS: PCOS with Metabolic Syndrome, aTL: Absolute telomere length, BMI: Body mass index, WC: waist circumference, SBP: Systolic blood pressure, DBP: Diastolic blood pressure, TC: Total cholesterol, HDL-C: High density cholesterol, LDL-C: Low density cholesterol, TG: Triglycerides, HOMA-IR: Homeostasis model assessment of insulin resistance, QUICKY: Quantitative Insulin Sensitivity Check Index. P<0.05 was considered as significant

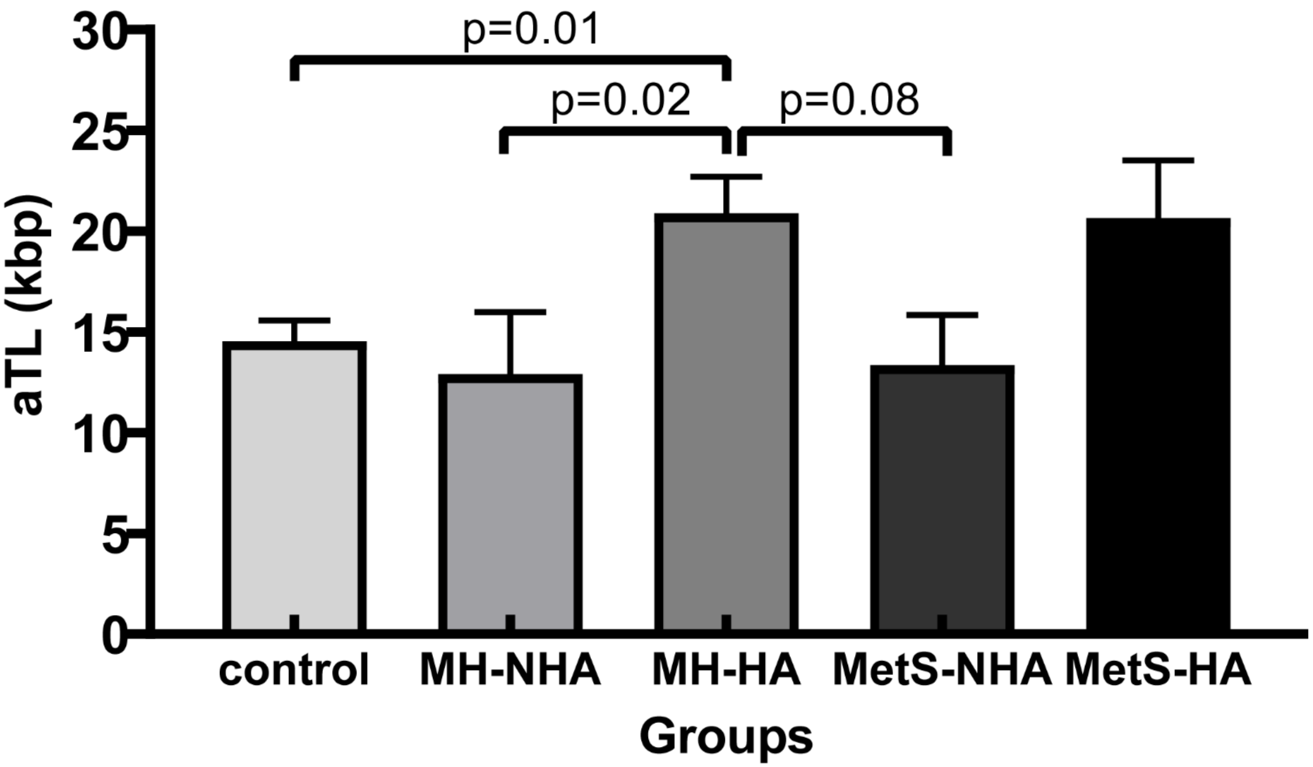

Supplementary Figure 1. Comparisons of aTL between control group and PCOS phenotypes HA and NHA with and without MetS. Values are presented as mean aTL and standard errors. aTL: Absolute telomere length. Kbp: kilobase pairs. MetS: Metabolic syndrome, MH-NHA: non hyperandrogenic and metabolically healthy PCOS women, MH-HA: Hyperandrogenic and metabolically healthy PCOS women; MetS-NHA: non hyperandrogenic PCOS with MetS; MetS-HA: Hyperandrogenic PCOS with MetS. Univariate analysis adjusted by age (ANCOVA). p<0.05 was considered as significant.
